# High proportions of post-exertional malaise and orthostatic intolerance in people living with post-COVID-19 condition: the PRIME post-COVID study

**DOI:** 10.3389/fmed.2023.1292446

**Published:** 2023-12-15

**Authors:** Demi M. E. Pagen, Maarten Van Herck, Céline J. A. van Bilsen, Stephanie Brinkhues, Kevin Konings, Casper D. J. den Heijer, Martijn A. Spruit, Christian J. P. A. Hoebe, Nicole H. T. M. Dukers-Muijrers

**Affiliations:** ^1^Department of Sexual Health, Infectious Diseases, and Environmental Health, Living Lab Public Health, South Limburg Public Health Service, Heerlen, Netherlands; ^2^Department of Social Medicine, Care and Public Health Research Institute (CAPHRI), Maastricht University, Maastricht, Netherlands; ^3^Department of Research and Education, Ciro, Horn, Netherlands; ^4^Department of Respiratory Medicine, School of Nutrition and Translational Research in Metabolism (NUTRIM), Maastricht University Medical Centre (MUMC+), Maastricht, Netherlands; ^5^Faculty of Rehabilitation Sciences, The Rehabilitation Research Center (REVAL), BIOMED–Biomedical Research Institute, Hasselt University, Diepenbeek, Belgium; ^6^Department of Knowledge and Innovation, South Limburg Public Health Service, Heerlen, Netherlands; ^7^Department of Process and Information Management, Communication and Automation, South Limburg Public Health Service, Heerlen, Netherlands; ^8^Department of Medical Microbiology, Care and Public Health Research Institute (CAPHRI), Maastricht University Medical Centre (MUMC+), Maastricht, Netherlands; ^9^Department of Health Promotion, Care and Public Health Research Institute (CAPHRI), Maastricht University, Maastricht, Netherlands

**Keywords:** COVID-19, long COVID, post-COVID-19 condition, post-exertional malaise, orthostatic intolerance

## Abstract

**Background:**

Exercise-based treatments can worsen/exacerbate symptoms in people who were SARS-CoV-2 positive and living with post-COVID-19 condition (PL-PCC) and who have post-exertional malaise (PEM) or orthostatic intolerance (OI). Nevertheless, PEM and OI are not routinely assessed by clinicians. We estimated PEM and OI proportions in PL-PCC, as well as in people not living with PCC (PnL-PCC) and negatives (i.e., never reported a SARS-CoV-2 positive test), and identified associated factors.

**Methods:**

Participants from the Prevalence, Risk factors, and Impact Evaluation (PRIME) post-COVID-19 condition study were included. PEM and OI were assessed using validated questionnaires. PCC was defined as feeling unrecovered after SARS-CoV-2 infection. Multivariable regression analyses to study PEM and OI were stratified for sex.

**Results:**

Data from 3,783 participants were analyzed. In PL-PCC, the proportion of PEM was 48.1% and 41.2%, and the proportion of OI was 29.3% and 27.9% in women and men, respectively. Proportions were higher in PL-PCC than negatives, for PEM in women OR=4.38 [95%CI:3.01–6.38]; in men OR = 4.78 [95%CI:3.13–7.29]; for OI in women 3.06 [95%CI:1.97-4.76]; in men 2.71 [95%CI:1.75–4.21]. Associated factors were age ≤ 60 years, ≥1 comorbidities, and living alone.

**Conclusion:**

High proportions of PEM and OI are observed in PL-PCC. Standard screening for PEM and OI is recommended in PL-PCC to promote appropriate therapies.

## 1 Introduction

There is an urgent need for information on optimal care and treatment options for people living with post-COVID-19 condition (PL-PCC) (1). PL-PCC are suffering from substantial, persistent symptoms after a SARS-CoV-2 infection. Experienced symptoms are very heterogenous, including fatigue, dyspnea, and cognitive dysfunctions, among others (2, 3). A link between post-COVID-19 condition (PCC) and myalgic encephalomyelitis or chronic fatigue syndrome (ME/CFS) has been made (4, 5), particularly based on the similarities in the presence or relapsing of unexplained symptoms, such as disabling fatigue, exhaustion, difficulty thinking, pain, exercise intolerance, and other symptoms (6, 7).

Post-exertional malaise (PEM) has previously been described in PCC (3, 8) and is a cardinal feature of ME/CFS (9, 10). PEM refers to the abnormal worsening of various symptoms (which can be fatigue) and loss of energy following minimal physical or cognitive stressors or other triggers that would have been tolerated normally before disease onset (6). PEM has been found to be more prevalent in women than in men (11), and infections can initiate PEM (12). Another comorbidity with ME/CFS is postural orthostatic tachycardia syndrome (POTS), which generally causes orthostatic intolerance (13). The majority of the POTS patients are women as well (14).

Rehabilitation of PL-PCC is often focused on applying exercise-based protocols, especially early on in the COVID-19 pandemic, as early reports of cases were derived from deconditioned hospitalized cases (8, 15). However, the relationship between physical activity and PCC is not well understood, with some studies describing improved symptoms and others describing symptom exacerbation (16). Furthermore, some PCC patients are offered cognitive behavioral therapy (CBT), but curative merit is criticized in line with concerns in ME/CFS patients (17). The presence of PEM or OI in people with PCC has important implications for their treatment options, as people can be intolerant to exercise, cognitive stressors, or upright position. There is evidence that exercise-based protocols can worsen/exacerbate symptoms (3, 18, 19). To date, the prevalence of PEM and OI in PL-PCC is not well known (20), but likely substantial.

This observational cohort study, called the Prevalence, Risk factors, and Impact Evaluation post-COVID study (PRIME post-COVID), estimated the proportion of PEM and OI in PL-PCC and people who were SARS-CoV-2 positive and not living with PCC (PnL-PCC) and adults who never reported a positive SARS-CoV-2 test (further referred to as negatives). Furthermore, we identified relevant subgroups that are more prone to have PEM or OI and described the occurrence of fatigue or other symptoms that may accompany PEM or OI in PL-PCC.

## 2 Materials and methods

### 2.1 Study design

The design and recruitment of the PRIME post-COVID study has been published previously (21). In brief, an observational open cohort study was set up with assessments of various health conditions and health factors. Invitees were adults tested for SARS-CoV-2 with a valid test result and email address, recorded in the public health registry in South Limburg, the Netherlands. The longitudinal character enabled additional data collection moments. After completing the baseline questionnaire (December 2021), participants were invited to participate in a follow-up questionnaire (August 2022).

### 2.2 Participants

In total, 12,453 initial participants were invited to complete the follow-up questionnaire. Data were collected using the online MWM2 application of market research platform Crowdtech (ISO 27001 certified). Participants who likely represented another person than the intended invitee (reported inconsistent information regarding sex and test result compared to the baseline questionnaire) were excluded.

### 2.3 Data collection

The follow-up questionnaire covered demographics (to construct variables on age, sex, level of education, and urbanity of living area), date and result of last COVID-19 test, physical health (height and weight to construct body mass index (BMI), and comorbidity), and smoking behavior.

Additionally, the questionnaire included the validated DePaul Symptom Questionnaire Post-Exertional Malaise (DSQ-PEM) (22) and four items from the DePaul Symptom Questionnaire-2 (DSQ-2) regarding OI (23, 24), and experienced symptoms (44 pre-listed) with severity scores (range 1–10). Based on the reported symptoms, participants were categorized into the following:

- Did not experience any symptoms now
- Experienced fatigue only
- Experienced fatigue and at least one other symptom
- Experienced multiple symptoms except fatigue

Frequencies and proportions of these categories were reported in PL-PCC with and without PEM or OI. The questionnaire further included a question of whether people felt recovered or not felt recovered since their first recorded SARS-CoV-2 infection.

### 2.4 Classification of COVID-19 test result

For people invited at baseline, COVID-19 test result was known in the national test registry. Additionally, participants self-reported SARS-CoV-2 infections in both questionnaires. Participants were classified as SARS-CoV-2 negatives when no positive test result was reported in both baseline (registry and self-report) and follow-up questionnaire (self-report). However, we have to acknowledge that at the time of the follow-up questionnaire (August 2022), the chance that people were truly negative and never had been infected before is small. Nevertheless, to retain the readability and clarity of this study, people who never reported a positive SARS-CoV-2 test will be referred to as negatives.

Participants who reported at least one positive test result (i.e., in baseline or follow-up) were classified as SARS-CoV-2 positive.

### 2.5 Outcome variables

#### 2.5.1 Post-exertional malaise

In the DSQ-PEM, respondents rated five items over the previous 6 months on frequency (never, sometimes, about half the time, most of the time, always) and severity (no, mild, moderate, severe, very severe) on a 5-point Likert scale. The five items were “A dead, heavy feeling after starting to exercise”, “Next day soreness or fatigue after non-strenuous, everyday activities”, “Mentally tired after the slightest effort”, “Minimum exercise makes you physically tired”, and “Physically drained or sick after mild activity”. A score on frequency of about half of the time to always and a score on severity of moderate to very severe on the same item on any of the five items is indicative of PEM (22). Additionally, in people who had PEM, a sum score (range 4–40; minimum of 4 due to the threshold for having PEM) of frequency (range 0–4) and severity (0–4) of the five items was calculated as severity measure (25).

#### 2.5.2 Orthostatic intolerance

OI was measured using four items selected from the DSQ-2. Respondents rated the four items over the previous 6 months on frequency (never, sometimes, about half the time, most of the time, always) and severity (no, mild, moderate, severe, very severe) on a 5-point Likert scale. The four items were “Rapid heartbeat after standing”, “Blurred or tunnel vision after standing”, “Gray or blacking after standing”, and “Inability to tolerate an upright position”. A score on the frequency of about half of the time to always and a score on the severity of moderate to very severe on the same item on any of the four items is indicative of OI. These four items were selected as they are used in the various classifications of ME/CFS to define OI (23, 24). In addition, in people who had OI, a severity sum score (range 4–32; minimum of 4 due to the threshold for having OI) was calculated based on frequency (range 0–4) and severity (range 0–4) of the four items.

Moreover, the co-occurrence of PEM and OI was described by reporting proportions of participants having: no PEM or OI; only OI; only PEM; and both PEM and OI.

### 2.6 Post-COVID condition definition and study population in current analyses

Several PCC definitions have previously been studied within the PRIME post-COVID study (26). In the current study, we aimed to inform clinicians about the proportions of PEM and OI in the PCC population as well as in the general population. As we sought to inform clinical practice, we considered it appropriate to use the PCC definition of not feeling recovered, as this most likely reflects the population who would present to medical care. Not feeling recovered has also been used as a PCC definition in various previous studies (26–32).

As sensitivity analyses, we presented various other PCC definitions and estimated PEM and OI proportions (26). These other PCC definitions included:

1. Having ≥1 of all 44 pre-listed symptoms
2. Having ≥1 symptoms that were significantly more often reported in positives than in negatives (in data of baseline questionnaire)
3. Having ≥1 of the selected symptoms in definition 2 AND with a severity score of ≥5 points (cutoff of 5 was used according to the mean of scores; range 1–10).

Besides, sensitivity analyses were performed by presenting PEM and OI proportions stratified for months since the first reported positive SARS-CoV-2 test (3–6, 6–9, 9–12, 12–18 and longer than 18 months ago) in PL-PCC using the PCC definition of not feeling recovered.

### 2.7 Associated factors

Several demographics (sex, age, level of education, and living alone), physical (obesity and comorbidities), lifestyle (current or former smoking behavior), and environmental factors (urbanity of living area) have been selected as factors possibly associated with PEM and OI. These subgroup characteristics are often known to the treating physician and might be of use to indicate high-prevalence subgroups. Age was dichotomized into 18–60 and 60+ age groups based on the age distribution of our study population. Level of education was categorized into people being practically (i.e., no, lower general, lower vocational, general secondary, and secondary vocational education) or theoretically (i.e., higher general, pre-university, higher professional, and scientific education) trained. Being obese was defined as having a BMI≥30 kg/m^2^. Urbanity of living area was based on postal code and categorized into: (very) strongly urban, moderately urban, little urban, and rural.

### 2.8 Statistical analysis

Participants who reported ME/CFS or fibromyalgia before their SARS-CoV-2 infection were excluded from the analyses to limit a possible risk of overestimating proportions of PEM and OI. People who tested SARS-CoV-2 positive <3 months before questionnaire completion were also excluded, because of the PCC definition window.

Other studies found that women more often had PEM and OI than men (11, 14). As this was confirmed in our study population for PEM, subsequent analyses were stratified by sex.

Proportions and 95% confidence intervals (CI) were calculated for PEM and OI in PL-PCC, PnL-PCC, and negatives. Associations with age, smoking behavior, living alone, urbanity of living area, obesity, or comorbidities were performed using multivariable logistic regression analyses. We also tested for effect modification between these factors and the PCC group. In these regression analyses, PnL-PCC were excluded. Independent-samples Mann–Whitney U test was used to test whether PEM and OI severity scores differed between PL-PCC and negatives. Analyses were performed using Statistical Package for Social Sciences (SPSS; version 27.0, IBM, Armonk, USA). A *p* < 0.05 was considered statistically significant.

### 2.9 Ethical statement and trial registry

The PRIME post-COVID study was waived by the Medical Ethical Committee of Maastricht University Medical Center+ (METC2021-2884). This study was registered at ClinicalTrials.gov Protocol Registration and Results System (NCT05128695).

## 3 Results

**Figure 1:**
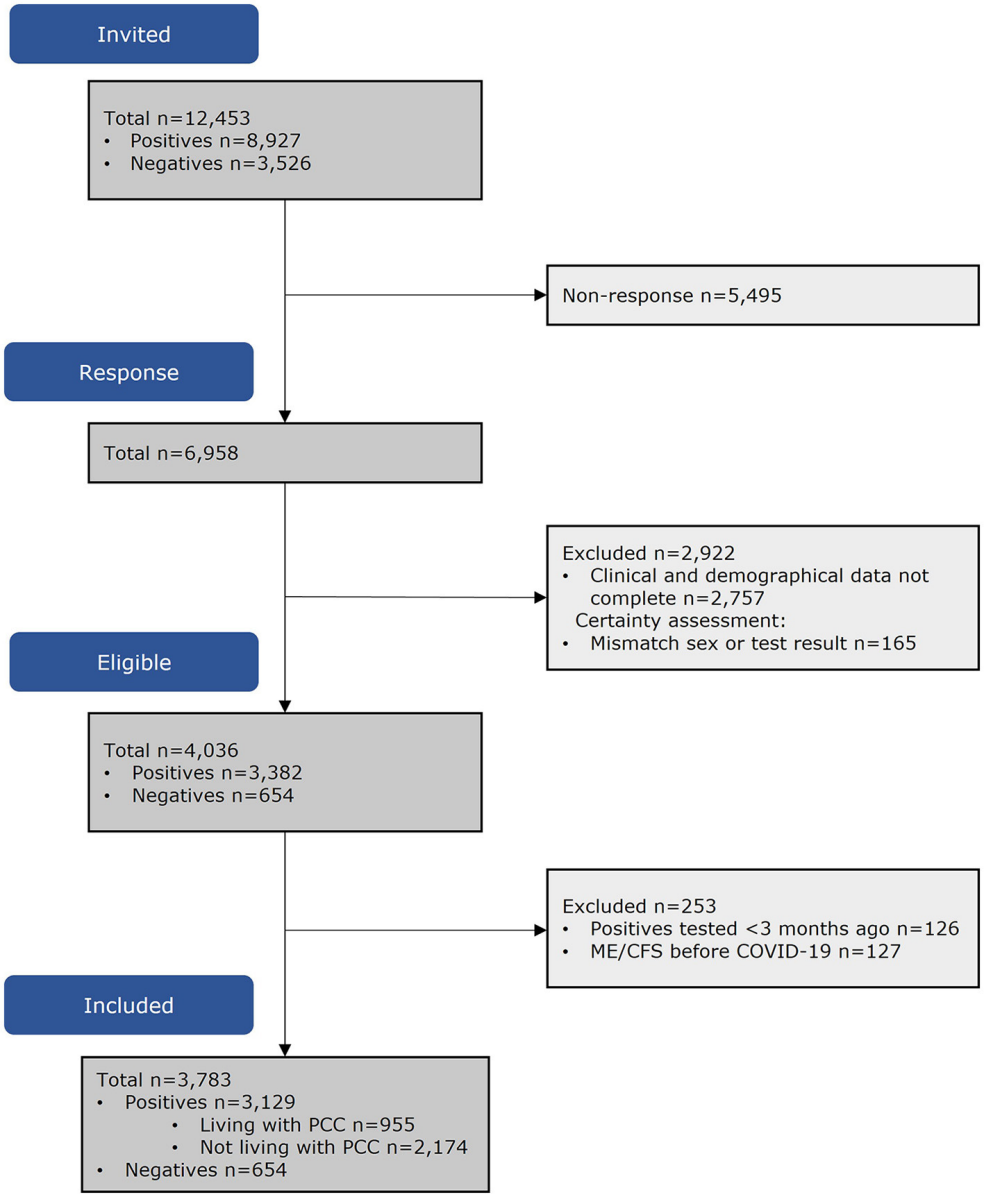
Flowchart of participants included in analyses.

Of the invitees (*n* = 12,453), 4,201 (60.4%) had complete data. Of the people who tested SARS-CoV-2 positive, 253 were excluded as they reported ME/CFS or fibromyalgia before SARS-CoV-2 infection or were tested <3 months before questionnaire completion. The population in analyses consisted of *n* = 955 PL-PCC, *n* = 2,174 PnL-PCC, and *n* = 654 negatives (Figure 1).

Groups differed regarding sex, age, educational level, BMI, comorbidities, and living alone (Table 1).

**Table 1:** Population characteristics, stratified for people who were SARS-CoV-2 positive and are living with post-COVID-19 condition (PL-PCC), people who were SARS-CoV-2 positive and not living with post-COVID-19 condition (PnL-PCC) and negatives.

	**PL-PCC (*n*** = **955)**		**PnL-PCC (*n*** = **2,174)**		**Negatives (*n*** = **654)**	
	**Men (*n* = 398)**	**Women (*n* = 557)**	**Men (*n* = 947)**	**Women (*n* = 1,227)**	**Men (*n* = 375)**	**Women (*n* = 279)**
Age, mean (SD)	58 (11)	54 (13)	57 (13)	53 (14)	65 (11)	60 (13)
18–60	266 (66.8)	419 (75.2)	573 (60.5)	881 (71.8)	138 (36.8)	151 (54.1)
60+	132 (33.2)	138 (24.8)	374 (39.5)	346 (28.2)	237 (63.2)	128 (45.9)
BMI, mean (SD)	28.3 (4.6)	27.5 (5.2)	27.2 (4.1)	26.2 (4.9)	26.7 (4.2)	26.6 (5.3)
Not obese	270 (67.8)	393 (70.6)	717 (75.7)	980 (79.9)	300 (80.0)	210 (75.3)
Obese	128 (32.2)	164 (29.4)	230 (24.3)	247 (20.1)	75 (20.0)	69 (24.7)
Comorbidities^*^, mean (SD)	0.93 (0.69)	0.91 (0.69)	0.58 (0.60)	0.53 (0.59)	0.69 (0.64)	0.74 (0.66)
0	109 (27.4)	160 (28.7)	452 (47.7)	637 (51.9)	154 (41.1)	106 (38.0)
1–2	207 (52.0)	287 (51.5)	439 (46.4)	527 (43.0)	185 (49.3)	139 (49.8)
>2	82 (20.6)	110 (19.7)	56 (5.9)	63 (5.1)	36 (9.6)	34 (12.2)
ME/CFS	12 (3.0)	5 (0.9)	0	0	1 (0.3)	2 (0.7)
Fibromyalgia	1 (0.3)	19 (3.4)	0	8 (0.7)	2 (0.5)	21 (7.5)
**Smoking** Never smoker							348 (87.4)	485 (87.1)	856 (90.4)	1,119 (91.2)	302 (80.5)	226 (81.0)
Former smoker	18 (4.5)	20 (3.6)	36 (3.8)	24 (2.0)	16 (4.3)	10 (3.6)
Current smoker	32 (8.0)	52 (9.3)	55 (5.8)	84 (6.8)	57 (15.2)	43 (15.4)
**Level of education** Practically trained							244 (61.3)	342 (61.4)	418 (44.1)	656 (53.5)	194 (51.7)	155 (55.6)
Theoretically trained	154 (38.7)	215 (38.6)	529 (55.9)	571 (46.5)	181 (48.3)	124 (44.4)
**Living alone** Yes							68 (17.1)	93 (16.7)	149 (15.7)	219 (17.8)	84 (22.4)	90 (32.3)
No	330 (82.9)	464 (83.3)	798 (84.3)	1,008 (82.2)	291 (77.6)	189 (67.7)
**Urbanity** (Very) strongly urban							140 (35.2)	199 (35.7)	347 (36.6)	464 (37.8)	141 (37.6)	108 (38.7)
Moderately urban	95 (23.9)	134 (24.1)	214 (22.6)	235 (19.2)	83 (22.1)	65 (23.3)
Little urban	100 (25.1)	122 (21.9)	235 (24.8)	297 (24.2)	86 (22.9)	64 (22.9)
Rural	63 (15.8)	102 (18.3)	151 (15.9)	231 (18.8)	65 (17.3)	42 (15.1)

BMI, body mass index; ME/CFS, myalgic encephalomyelitis or chronic fatigue syndrome; PnL-PCC, people who were SARS-CoV-2 positive and not living with post-COVID-19 condition; PL-PCC, people who were SARS-CoV-2 positive and living with post-COVID-19 condition.

^*^Comorbidities reported when completing follow-up questionnaire, ME/CFS and fibromyalgia not included but reported separately.

For negatives, it was not known whether ME/CFS and fibromyalgia diagnosis was known before or after SARS-CoV-2 testing.

### 3.1 Proportion estimates of PEM

**Figure 2:**
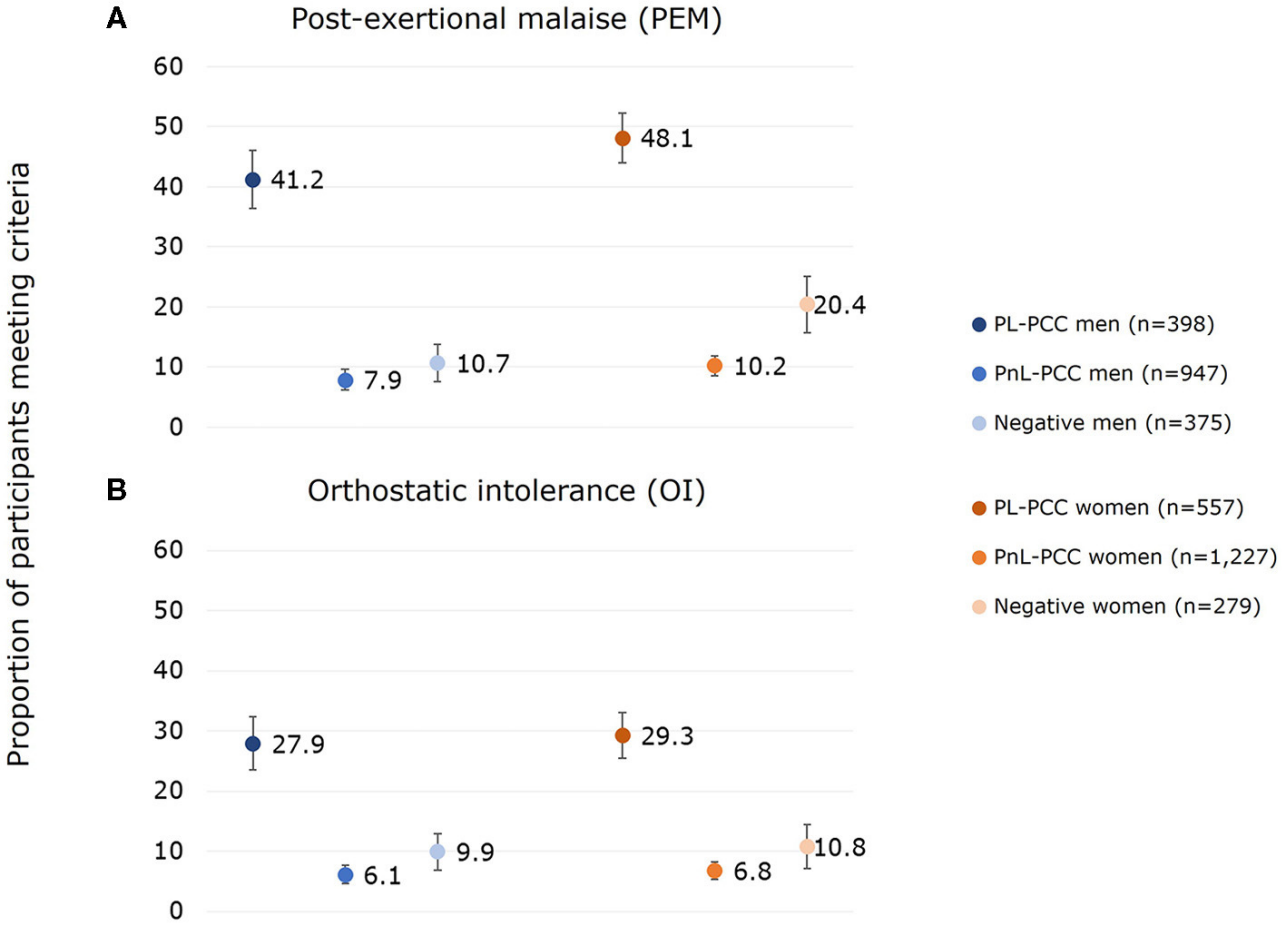
Proportion of **(A)** post-exertional malaise (PEM) and **(B)** orthostatic intolerance (OI) for men and women who had PEM or OI and who were SARS-CoV-2 positive and not living with post-COVID-19 condition (PnL-PCC), SARS-CoV-2 positive and living with post-COVID-19 condition (PL-PCC), and negatives.

The proportion of PEM in all positives was 23.2% (95% CI:21.2%−25.2%) in women and 17.8% (95% CI:15.8%−19.8%) in men. The proportion of PEM was 48.1% in PL-PCC women (95% CI:44.0%−52.2%) and was lower (*p* = 0.035) in PL-PCC men, with a proportion of 41.2% (95% CI:36.4%−46.0%) (Figure 2A). The proportion in negatives was 20.4% (95% CI:15.7%−25.1%) in women and 10.7% (95% CI:7.6%−13.8%) in men (*p* < 0.001), and the proportion in PnL-PCC was 10.2% (95% CI:8.5%−11.9%) in women and 7.9% (95% CI:6.2%−9.6%) in men (*p* = 0.066). The proportion of PEM in PL-PCC was between 38.6% and 47.8% for women and between 31.8% and 41.4% for men when using other PCC definitions (Supplementary Figure 1). The proportion of PEM in PL-PCC ranged from 39.1% to 56.8% in women and from 38.9% to 52.5% in men during the various periods since testing (Supplementary Table 1).

PL-PCC women had 4.38 (95% CI:3.01–6.38) higher odds of having PEM than negative women after adjusting for age, level of education, smoking behavior, living alone, urbanity of living area, obesity, and comorbidities. For PL-PCC men, the adjusted odds ratio (OR) was 4.78 (95% CI:3.13–7.29).

### 3.2 Proportion estimates of OI

The proportion of OI in all positives was 13.8% (95% CI:12.2%-15.4%) in women and 12.6% (95% CI:10.8%-14.4%) in men. The proportion of OI was 29.3% (95% CI:25.5%-33.1%) in PL-PCC women and 27.9% in PL-PCC men (95% CI:23.5%-32.3%) (p=0.638) (Figure 2B). The proportion in negatives was 10.8% (95% CI:7.2%-14.4%) in women and 9.9% (95% CI:6.9%-12.9%) in men (p=0.711), and the proportion in PnL-PCC was 6.8% (95% CI:5.4%-8.2%) in women and 6.1% (95% CI:4.6%-7.6%) in men (p=0.509). The proportion of OI in PL-PCC was between 22.4% and 29.1% for women and between 20.4% and 27.9% for men when using other PCC definitions (Supplementary Figure 1). The proportion of OI in PL-PCC ranged from 19.6% to 35.1% in women and from 21.4% to 32.5% in men by the various periods since testing (Supplementary Table 1).

PL-PCC women had 3.06 (95% CI:1.97–4.76) higher odds of having OI than negative women after adjusting. For PL-PCC men, the adjusted OR was 2.71 (95% CI:1.75–4.21).

### 3.3 Co-occurrence between PEM and OI

**Figure 3:**
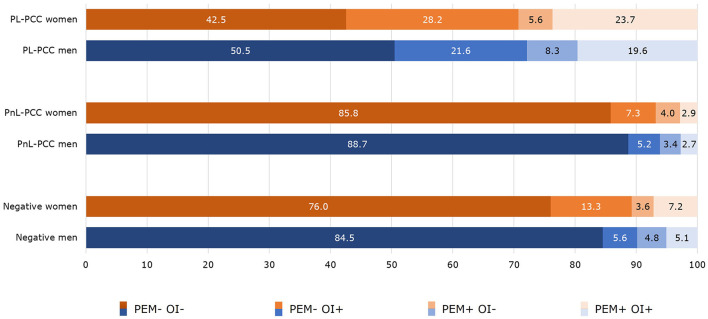
Co-occurrence of post-exertional malaise (PEM) and orthostatic intolerance (OI) in people who were SARS-CoV-2 positive and living with post-COVID-19 condition (PL-PCC), people who were SARS-CoV-2 positive and not living with post-COVID-19 condition (PnL-PCC) and negatives.

PEM and OI were co-occurrent in 19.6% of the PL-PCC men and 23.7% of the PL-PCC women. For PnL-PCC, PEM and OI were co-occurrent in 2.7% for men and 2.9% for women. In negatives, this was 5.1% in men and 7.2% in women (Figure 3).

### 3.4 Severity score of PEM and OI

**Figure 4:**
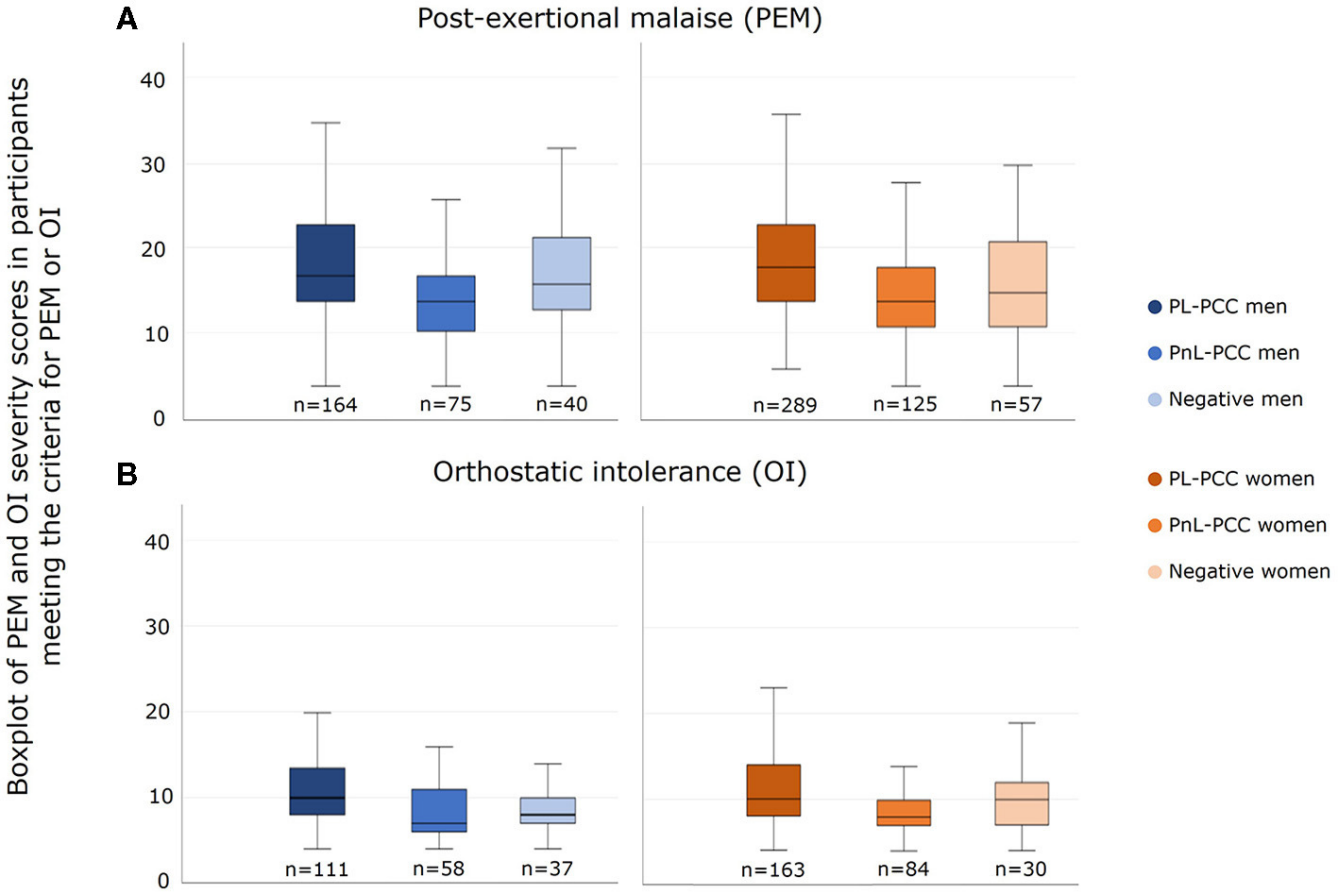
Severity score of **(A)** post-exertional malaise (PEM) and **(B)** orthostatic intolerance (OI) in men and women who had PEM or OI and who were SARS-CoV-2 positive and not living with post-COVID-19 condition (PnL-PCC), SARS-CoV-2 positive and living with post-COVID-19 condition (PL-PCC), and negatives.

In people who had PEM, the median PEM severity score (range 4–39) in PL-PCC was 17 in women and 18 in men. In PnL-PCC, the median PEM score was 14 in both men and women. In negatives, the median PEM score was 15 in women and 16 in men. The median PEM score was higher in PL-PCC women than negative women (p=0.003) (Figure 4A).

In people who had OI, the median OI severity score (range 4–27) in PL-PCC was 10 in both men and women. In PnL-PCC, the median OI score was 8 in women and 7 in men. In negatives, the median OI score was 10 in women and 8 in men. The median OI severity score was higher in PL-PCC men than in negative men (*p* = 0.003) (Figure 4B).

### 3.5 Associated factors for PEM and OI

In both men and women, factors associated with a higher risk for PEM were being 60 years or younger, living alone, having at least one comorbidity, and smoking (women only) (Table 2).

**Table 2:** Factors associated with post-exertional malaise (PEM) or orthostatic intolerance (OI) in people who were SARS-CoV-2 positive and living with post-COVID-19 condition (PL-PCC) and negatives in multivariable regression analyses.

	**Post-exertional malaise (PEM)**								**Orthostatic intolerance (OI)**							
	**Men (*****n*** = **773)**				**Women (*****n*** = **836)**				**Men (*****n*** = **773)**				**Women (*****n*** = **836)**			
	**PEM (%)**	**OR**	**95% CI**		**PEM (%)**	**OR**	**95% CI**		**OI (%)**	**OR**	**95% CI**		**OI (%)**	**OR**	**95% CI**	
PL-PCC (negatives ref.)		4.78^†^	3.13	7.29		4.38^†^	3.01	6.38		2.71^†^	1.75	4.21		3.06^†^	1.97	4.76
**Age**																
60+ (ref.)	43.6	1.00			53.7	1.00			31.6	1.00			30.3	1.00		
18-60	36.4	1.70^*^	1.16	2.51	46.4	1.99^†^	1.39	2.86	20.5	1.87^*^	1.23	2.83	26.1	1.46	0.98	2.18
**Level of education**																
Theoretically trained (ref.)	42.6	1.00			55.8	1.00			27.9	1.00			30.7	1.00		
Practically trained	39.0	1.05	0.72	1.53	45.6	1.29	0.93	1.78	27.9	0.95	0.64	1.42	27.0	1.20	0.83	1.72
**Smoking behavior**																
Never smoker (ref.)	41.7	1.00			49.5	1.00^*^			27.6	1.00			27.8	1.00		
Former smoker	50.0	1.99	0.89	4.48	60.0	1.78	0.79	4.04	27.8	1.35	0.58	3.16	45.0	1.97	0.86	4.51
Current smoker	31.1	1.23	0.69	2.22	71.2	2.12^*^	1.29	3.50	31.3	1.1	0.59	2.05	36.5	1.57	0.94	2.63
**Living alone**																
No (ref.)	38.5	1.00			50.0	1.00			25.5	1.00			30.0	1.00		
Yes	54.4	1.62^*^	1.03	2.54	61.3	1.73^*^	1.16	2.57	39.7	1.60^*^	1.01	2.55	25.8	0.78	0.50	1.22
**Urbanity of residential area**																
Rural (ref.)	36.5	1.00			41.2	1.00			20.6	1.00			11.0	1.00		
Little urban	33.0	0.85	0.46	1.54	52.5	1.66^*^	1.00	2.74	25.0	1.38	0.71	2.69	23.3	1.84^*^	1.03	3.28
Moderately urban	47.4	1.45	0.80	2.62	55.2	1.52	0.93	2.49	31.6	1.6	0.82	3.16	27.6	1.83^*^	1.04	3.22
(Very) strongly urban	45.0	1.03	0.59	1.80	54.8	1.55	0.98	2.47	30.7	1.47	0.78	2.76	38.0	1.64	0.95	2.81
**Obese**																
No (ref.)	37.0	1.00			48.9	1.00			25.2	1.00			27.5	1.00		
Yes	50.0	1.27	0.85	1.90	59.1	1.41	1.00	2.00	33.6	1.09	0.71	1.67	33.5	1.20	0.83	1.75
**Comorbidities**																
0 (ref.)	25.7	1.00^†^			40.0	1.00^†^			22.9	1.00^†^			24.4	1.00^*^		
1-2 comorbidities	42.5	3.19^†^	2.00	5.10	53.3	2.44^†^	1.69	3.51	25.1	1.96^*^	1.21	3.17	28.9	1.69^*^	1.11	2.57
>2 comorbidities	58.5	6.61^†^	3.70	11.83	65.5	4.15^†^	2.55	6.75	41.5	3.98^†^	2.22	7.16	37.3	2.46^*^	1.47	4.14

CI, confidence interval; OR, odds ratio; ref., reference group.

PEM and OI proportions were determined in people who were SARS-CoV-2 positive and living with post-COVID-19 condition (PL-PCC), men n = 398, women n = 557.

^†^p <0.001; ^*^p <0.05.

In both men and women, factors associated with a higher risk for OI were having at least one comorbidity, while being 60 years or younger and living alone were associated factors in men only (Table 2).

Effect modification was observed between the PCC group (PL-PCC vs. negatives) and comorbidities, namely for both PEM and OI, the adjusted OR for comorbidities in PL-PCC was lower than in negatives (though statistically significant in both groups). Effect modification was observed between the PCC group and smoking for PEM, where smoking was associated in negative men (OR_former smoker_ = 5.04 [95%CI:1.62–15.65]; OR_current smoker_ = 2.36 [95%CI:1.06–5.23]), but not in PL-PCC men (OR_former smoker_ = 1.40 [95%CI:0.54–3.61]; OR_current smoker_ = 0.64 [95%CI:0.29–1.38]).

### 3.6 Experiencing fatigue in PL-PCC

**Figure 5:**
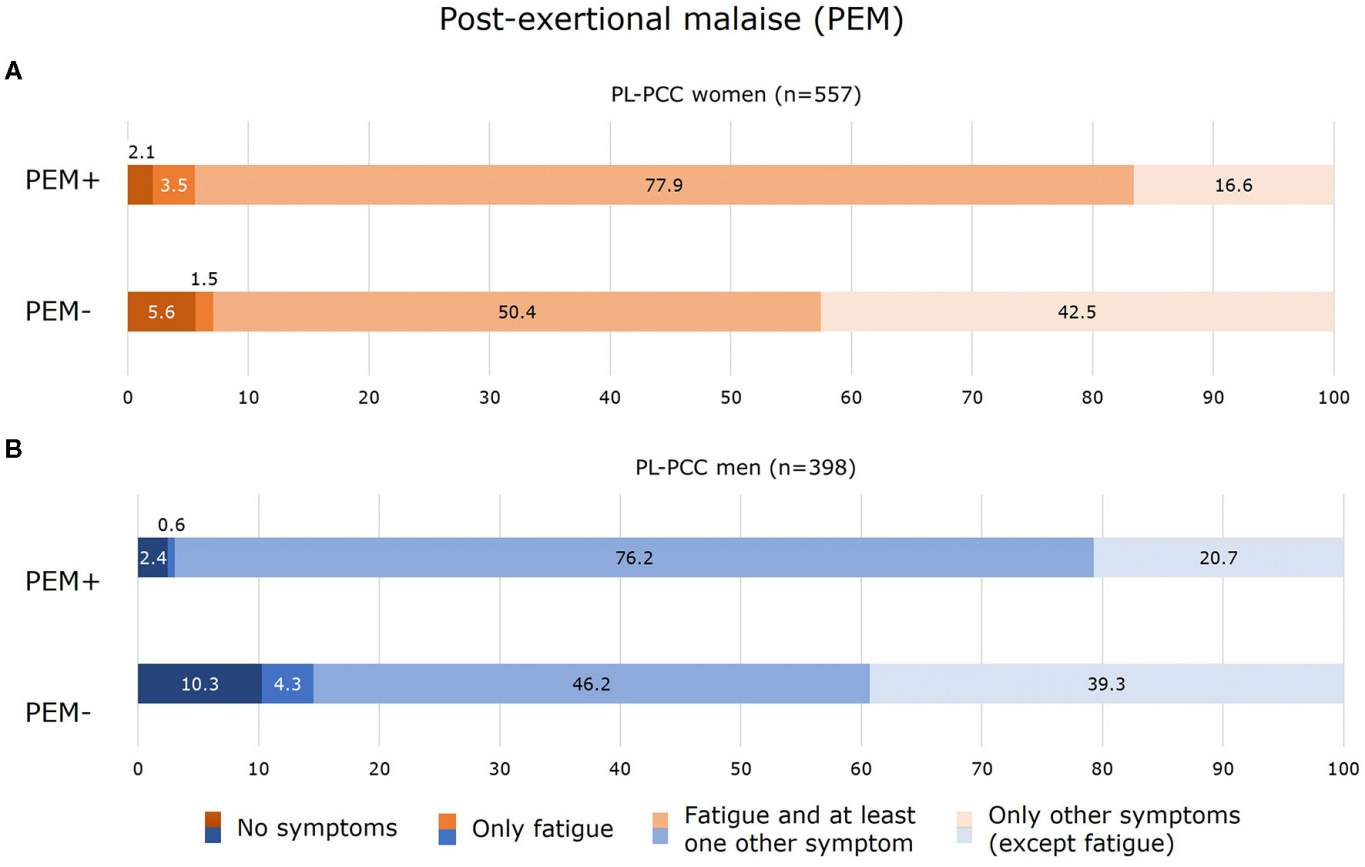
Experienced symptoms for women **(A)** and men **(B)** who were SARS-CoV-2 positive and living with post-COVID-19 condition (PL-PCC) with post-exertional malaise (PEM+) and without PEM (PEM−).

Of the PL-PCC women who had PEM, 81.3% currently experienced fatigue (of which the majority also had other symptoms) (Figure 5A). In PL-PCC men who had PEM, 76.8% experienced fatigue (the majority also had other symptoms); thus about one in five PL-PCC with PEM did not report fatigue (Figure 5B).

**Figure 6:**
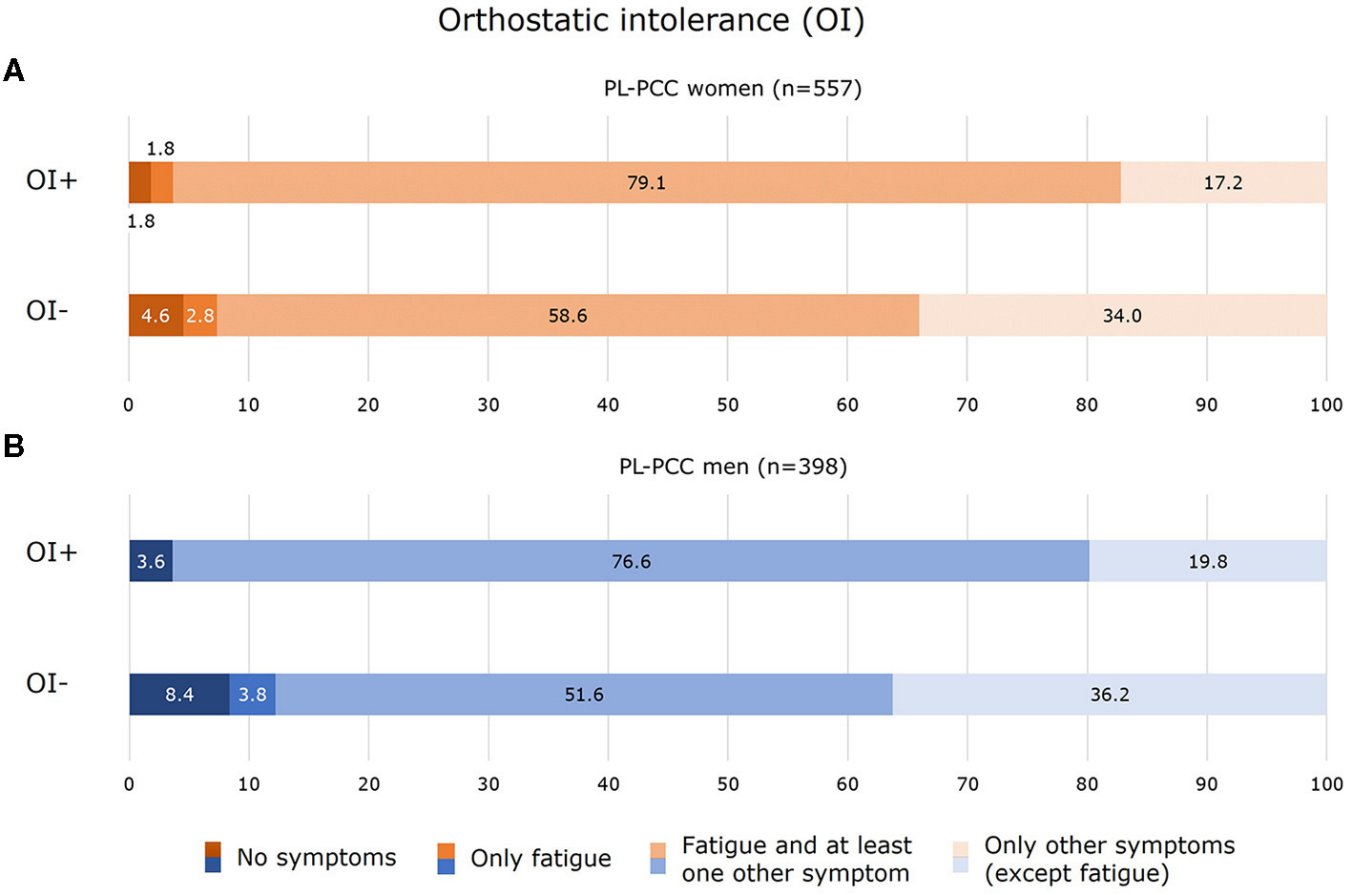
Experienced symptoms for women **(A)** and men **(B)** who were SARS-CoV-2 positive and living with post-COVID-19 condition (PL-PCC) with orthostatic intolerance (OI+) and without OI (OI−).

Of the PL-PCC women who had OI, 81.0% currently experienced fatigue (of which the majority also experienced other symptoms) (Figure 6A). In PL-PCC men who had OI, 76.6% experienced fatigue and other symptoms (Figure 6B).

## 4 Discussion

The results of the PRIME post-COVID study demonstrate that of people who feel unrecovered since SARS-CoV-2 infection (i.e., defined as PL-PCC), between 48.1% (women) and 41.2% (men) have PEM. Although PEM and OI are identified frequently in both men and women, PEM is more prevalent in PL-PCC women. The proportion of OI is comparable between PL-PCC men (27.9%) and women (29.3%). Of PL-PCC, PEM and OI were concurrent in 19.6% and 23.7% of men and women, respectively. Proportions of PEM and OI were notably higher in people of middle or younger age, those with comorbidities, and those living alone. The high proportions of PEM and OI call for standard screening in PL-PCC, regardless of whether fatigue is reported, by medical and allied healthcare professionals to avoid inappropriate exercise-based treatment for PL-PCC.

A previous study showed OI estimates to be comparable to the current study with 30.7%, but PEM estimates in PL-PCC were higher with 81.9% (3). The PEM proportion estimates of the current study are substantially lower (between 41.2% and 48.1%). This might (partly) be explained by different recruitment methods. The current study invited adults being tested and registered in the national COVID-19 registry, thereby recruiting a population-based sample. The previous study partly recruited participants via COVID-19 online support groups, probably including a high proportion of more severe or more aware PCC cases, resulting in selection bias. Besides, a greater proportion of women (78.9%) was included compared with the current study (58.3%), overestimating PEM proportions as PEM is more prevalent in women than men. Furthermore, items used to define PEM differed, as the previous study used only one item. Another study estimated PEM prevalence to be 58.7% in adults experiencing persistent symptoms (≥4 weeks) since infection, using the same validated DSQ-PEM questionnaire (33). They also included a higher share of women (85.5%); however, the prevalence of PEM was more comparable to the results of the current study. As the prevalence of both PEM and OI in the general population is not well known, including a SARS-CoV-2 negative group (as in our study) to estimate background risk is recommended.

The proportions of PEM and OI in negatives were 10.7%−20.4% and 9.9%−10.8%, respectively. Compared with negatives, the PEM and OI proportions were substantially higher in PL-PCC regardless of the PCC definition applied (Supplementary Figure 1). Notably, the proportion of PEM was higher in negative women (20.4%) than in PnL-PCC women (10.2%; *p* = 0.003). The reason is unknown; however, an explanation might be possible misclassification regarding being truly SARS-CoV-2 negative, as we could not rule that out initially, SARS-CoV-2 tested negative people might have been untested and actually be SARS-CoV-2 positive. In addition, the PnL-PCC group explicitly mentioned that they felt recovered since the infection, which probably resulted in a lower chance of having PEM. In the negatives, we did not ask whether they felt recovered after infection, as they did not report a positive SARS-CoV-2 test. Furthermore, we were unable to exclude negatives who already had ME/CFS or fibromyalgia before their SARS-CoV-2 test, which we were able to do for the positives.

The current study revealed that people with comorbidities or those who are living alone had substantially more often PEM or OI, which calls for specific attention to these subgroups when presenting with PCC. Nevertheless, in all subgroups, proportion estimates were higher in PL-PCC than in negatives.

Our results should alert clinicians and allied healthcare professionals to standardly screen for PEM and OI in people who feel unrecovered after SARS-CoV-2 infection or in those who might have PCC. Report of fatigue is not a suitable indicator for PEM or OI to be used in practice (i.e., fatigue is not reported in about one in five PL-PCC who have PEM or OI). Screening should thus be done using appropriate tools. Fortunately, there are simple and easy-to-use questionnaires available for healthcare professionals to use in daily practice. The DSQ-PEM and DSQ-2 are freely available and composed of only a few questions to validly indicate the presence of PEM or OI. These questionnaires and corresponding cutoff values are currently used in daily practice when diagnosing ME/CFS.

Some strengths and limitations of our study must be discussed. First, the large population-based cohort, including SARS-CoV-2 negatives, represents the main strength. Until now, studies only included PL-PCC when estimating PEM and OI proportion, without using a SARS-CoV-2 reference group. Second, validated questionnaires were used to assess PEM and OI. Third, we were able to demonstrate that the PEM and OI proportions in PL-PCC were more or less similar when using different PCC definitions, or when examining different periods since infection, stating the robustness of the proportion estimates.

The main limitation is the possibility of selection bias. Only 56% of invitees participated in the follow-up questionnaire. It is unknown whether non-participation has led to selection bias and over- or underestimation of PEM and OI. Furthermore, misclassification regarding the negative tested group being potentially SARS-CoV-2 positive cannot be ruled out. In such case, part of the observed proportions of PEM and OI in the negative tested group might be attributable to SARS-CoV-2 infection. Such misclassification is possible since infections might be missed due to limited testing possibilities at the beginning of the pandemic, lack of a reason to seek testing (e.g., asymptomatic cases), or a lack of testing intention or non-feasibility. Furthermore, it is likely that people with the most severe PCC, PEM, and OI were not included in the questionnaire, since they would be unable to complete the relatively long set of questions. This may cause an underestimation of the severity and proportion of PEM and OI in people living with PCC by the current study.

In conclusion, more attention and better identification of PEM and OI in PL-PCC are urgently needed to tailor treatment strategies, avoid exercise-based treatments that worsen/exacerbate symptoms, and promote appropriate and safe therapies. Therefore, we suggest standard screening for PEM and OI to increase identification by healthcare professionals by using simple questionnaires. Furthermore, special attention should be given to people having comorbidities or living alone.

## Data availability statement

The datasets presented in this article are not readily available because the data contains potentially identifying patient information. Data are available on request from the head of the data-archiving South Limburg Public Health Service for researchers who meet the criteria for access to confidential data. Requests to access the datasets should be directed to Helen.Sijstermans@ggdzl.nl.

## Ethics statement

The PRIME-post COVID study was waived by the Medical Ethical Committee of Maastricht University Medical Centre+ (METC2021-2884). This study was registered at ClinicalTrials.gov Protocol Registration and Results System (NCT05128695). The participants provided their written informed consent to participate in this study.

## Author contributions

DP: Conceptualization, Formal analysis, Methodology, Project administration, Visualization, Writing – original draft, Writing – review & editing. MV: Conceptualization, Methodology, Writing – review & editing. CB: Conceptualization, Investigation, Methodology, Project administration, Writing – review & editing. SB: Conceptualization, Investigation, Methodology, Project administration, Supervision, Writing – review & editing. KK: Conceptualization, Data curation, Project administration, Writing – review & editing. CHe: Conceptualization, Methodology, Supervision, Writing – review & editing. MS: Conceptualization, Methodology, Writing – review & editing. CHo: Conceptualization, Methodology, Resources, Supervision, Writing – review & editing. ND-M: Conceptualization, Funding acquisition, Methodology, Resources, Supervision, Writing – review & editing.
